# Association of Hearing Loss With HIV in Children

**DOI:** 10.7759/cureus.80022

**Published:** 2025-03-04

**Authors:** Deepika Acharya, Alok Hemal, Chetan S Tanwar

**Affiliations:** 1 Department of Pediatrics, Vardhman Mahavir Medical College and Safdarajung Hospital, New Delhi, IND; 2 Pediatrics, Dr. Ram Manohar Lohia Hospital, Delhi, IND; 3 Pediatrics, Sachkhand Hospital, Neemrana, IND

**Keywords:** hearing loss, hiv infection, otoscopy, pure-tone audiometry, viral load

## Abstract

Objectives: The study aimed to assess the incidence of hearing loss (HL) in children with human immunodeficiency virus (HIV) using pure-tone audiometry and to find the association of HL with the duration of the disease and severity of HIV infection.

Methods: A cross-sectional study was done on 60 children in the age group of five to 18 years who presented to the anti-retroviral therapy (ART) clinic for follow-up. All children were confirmed cases of HIV by reverse transcription polymerase chain reaction (RT-PCR). For the study, all HIV-positive children were screened for any HL. Pure-tone audiometry was used for determining the hearing test. The degree and type of HL were assessed. The laterality of HL was assessed. The association of HL with the CD4 count and viral load was assessed. Mann-Whitney test, Fisher's exact test, and independent t-test were used for statistical analysis with a p-value < 0.05 being considered statistically significant.

Results: HL was present in 10 (16.67%) cases, and all of them had conductive HL. Six cases had unilateral HL, while four cases had bilateral HL. In the majority (seven (70.00%)) of patients, the degree of HL was mild, followed by moderate HL (two (20.00%)) and minimal in only one (10.00%) patient. Compared to the HL- group, the HL+ group had significantly lower mean CD4 counts (483 ± 247.17 vs. 690.32 ± 301.09, p-value = 0.046) and significantly higher median viral load (2036.5 vs. 0, p < 0.0001). A significantly strong positive correlation was seen between the severity of HL with the viral load (r = 0.749, p = 0.018), and a significantly very strong negative correlation was seen between the severity of HL with CD4 counts (r = -0.809, p = 0.008).

Conclusion: An incidence rate of 16.67% HL was found among children with HIV. HL was predominantly mild in nature, and all cases had conductive HL. HL holds a direct correlation with increasing severity of HIV disease and subsequent falling of CD4 counts. Based on the results, it is recommended that all HIV-positive children must undergo routine audiology screening to determine the HL and provide them with audiological aid.

## Introduction

Human immunodeficiency virus (HIV) can cause significant complications, among which hearing loss (HL) may be a significant concern seen in 29-44% children [1,2] and in adults [3]. HL can be of three types in the form of conductive HL (CHL), sensorineural HL (SNHL), and mixed HL [1]. All these three types of HL can occur in HIV-infected individuals due to a direct effect of the virus or the use of ototoxic drugs or by the occurrence of opportunistic actions or by a direct action of the virus on the auditory system [1,2].

Other symptoms that may be seen along with HL include otorrhoea, otalgia, vertigo, and sensation of buzzing in the ear [1,2].

HL is widespread among children worldwide, affecting approximately 34 million children, with 60% of these cases resulting from causes that could have been prevented. HL, especially in young children, can result in delays in the development of speech and spoken language skills [4,5]. Hearing impairment can impact not only physical and mental health but also the overall quality of life. In addition, HL can negatively influence a child's educational progress, social interactions, and cognitive abilities [6].

It is known that HIV increases the risk of both peripheral and central HL in affected children by disrupting normal auditory processing. The most prevalent type of HL is CHL in children, which is primarily caused by ear infections such as otitis media. In the case of SNHL associated with HIV infection, the underlying mechanisms involve the impact of the disease and certain medications on mitochondrial function, leading to ototoxicity in susceptible individuals. In addition, exposure to other ototoxic substances, like aminoglycosides, along with noise exposure and infections such as meningitis and tuberculosis, further elevate the risk of HL in HIV-infected children [7].

There are only few studies in the Western world, which shows significant HL in HIV patients, which emphasizes the need for periodic hearing assessment in routine clinical care of HIV-infected children - thus favoring adequate development in language and decreasing possible difficulties in learning and social inclusion [8-12]. There is paucity of Indian data about HL in HIV-positive children. Thus, the present study was done to assess the incidence of HL in HIV-positive children, using pure-tone audiometry. The study also aimed to find the association of HL with the duration of disease and severity of HIV infection.

## Materials and methods

A cross-sectional study was done on 60 children in the age group of five to 18 years who presented to the anti-retroviral therapy (ART) clinic for follow-up at Atal Bihari Vajpayee Institute of Medical Sciences (ABVIMS), Dr. Ram Manohar Lohia Hospital, a tertiary care hospital in New Delhi over a period of January 1, 2021 to May 31, 2022. Any child lying in the age group of five to 18 years with a confirmed diagnosis of HIV on RT-PCR was included in the study. The exclusion criteria were children with attention-deficit hyperactivity disorder (ADHD), involuntary muscle movements (chorea), and other conditions that affect concentration; congenital anomalies like microtia, microcephaly, and syndromes like Wadenberg, Treacher Collins syndrome; actively draining ears (infected); comorbid conditions like head fracture; and terminal illness. For the study, all HIV-positive children were screened for any HL in them.

Sample size calculation 

In a study by Nakku et al. [7], it was found that the prevalence rate of HL in HIV-positive children was 8.8%. Using this prevalence rate as a reference, a minimum sample size of 55 patients was calculated, considering a 7.5% margin of error and a 5% level of significance. To further decrease the margin of error, the sample size was increased to 60.

Based on the eligibility criteria and sample size calculation, 60 children were included in the study. A written informed consent was obtained from the guardians or caregiver or parents. Institutional ethical clearance from the Institutional Ethical Committee of Atal Bihari Vajpayee Institute of Medical Sciences (ABVIMS), Dr. Ram Manohar Lohia Hospital, New Delhi, was obtained before beginning the study (F. No. TP (MD/MS) (91/2020)/ IEC/ ABVIMS/ RMLH).

Methodology

Demographic and clinical details of the patients were noted in terms of age, gender, age at diagnosis of HIV infection, duration of HIV, ART drugs, past clinical history, and clinical features. Symptoms were noted, and blood samples were taken to determine the viral load and for doing CD4 counts.

For ear examination, otoscopy was done to rule out any active ear infection. Pure-tone audiometry was used for determining the hearing test, which was carried out in an ambient room. Degree and type of HL was assessed. The laterality of HL was assessed. Association of HL with these parameters was assessed.

Classification

The HL was classified in terms of decibels of HL, where up to 25 decibels of HL was considered a normal and more than 80 was considered as profound HL. Any HL between 26 and 40 was labeled as slight or minimal, 41 to 60 as moderate, and 61 to 80 as severe HL. The HL was labeled as CHL if the airborne gap was present and sensory neural HL, if no airborne gap was present.

Outcomes

The outcome measures were the prevalence of HL in HIV patients and its association with the viral load and CD4 count.

Statistical analysis

Categorical variables were expressed as frequencies and percentages. Quantitative data were reported as means ± standard deviation (SD) or medians with interquartile ranges (25th-75th percentiles). The Shapiro-Wilk test assessed data normality, and non-parametric tests were applied when normality was not met. 

The association of the variables, which were quantitative and not normally distributed in nature, were analyzed using Mann-Whitney test, and variables, which were quantitative and normally distributed in nature, were analyzed using independent t-test. Fisher’s exact test was used for qualitative variables when the expected cell counts were below five. Spearman rank correlation coefficient was used to assess the correlation of HIV severity with the severity of HL.

Data were entered in Microsoft Excel (Microsoft Corp., Redmond, USA) and analyzed using IBM SPSS Statistics for Windows, version 25.0 (released 2017, IBM Corp., Armonk, NY), with statistical significance set at p < 0.05.

## Results

The mean age of the enrolled children were 12.61 ± 2.9 years. Regarding gender distribution, 34 (56.67%) cases were males and 26 (43.33%) cases were females. The mean age at diagnosis was 5.12 ± 3.18 years. Among the participants, 36 (60%) cases were diagnosed between one and five years, 21 (35%) cases were diagnosed between six and 10 years, and three (5%) cases were diagnosed between 11 and 15 years. The mean duration of HIV was 7.38 ± 3.18 years (Table 1).

**Table 1:** Demographic and clinical characteristics of the children

Parameters	Mean + -SD	N (%)
Age, y	12.61 ± 2.9	Not applicable
Males	Not applicable	34 (56.67%)
Females	Not applicable	26 (43.33%)
Age at diagnosis, y	5.12 ± 3.18	Not applicable
Duration of HIV, y	7.38 ± 3.18	Not applicable

The majority of patients (31.67%) in our study were on the zidovudine, lamivudine, and nevirapine (ZLN) regimen. A total of 16 patients (26.67%) were on the zidovudine, lamivudine, and efavirenz (ZLE) regimen, while nine patients (15.00%) were on the abacavir, lamivudine, and lopinavir/ritonavir (AL l/r) regimen.

**Figure 1:**
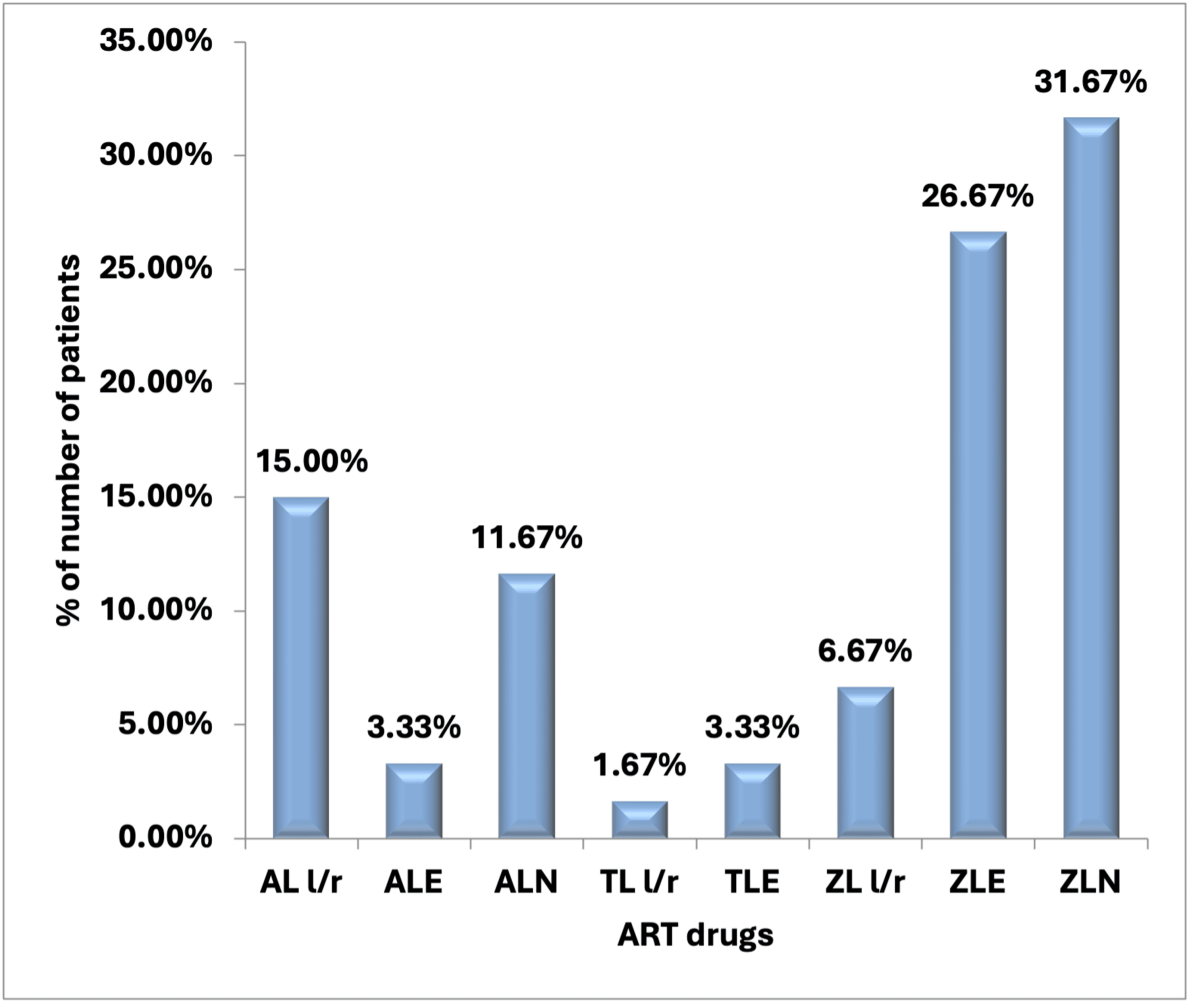
Distribution of ART drugs of the study subjects ART: antiretroviral therapy, ZLN: zidovudine, lamivudine, and nevirapine, ZLE: zidovudine, lamivudine, and efavirenz, AL l/r: abacavir, lamivudine, and lopinavir/ritonavir, ALN: abacavir, lamivudine, and nevirapine, ZL l/r: zidovudine, lamivudine, and lopinavir/ritonavir, ALE: abacavir, lamivudine, and efavirenz, TLE: tenofovir, lamivudine, and efavirenz, TL l/r:  tenofovir, lamivudine, and lopinavir/ritonavir

In addition, seven patients (11.67%) were on the abacavir, lamivudine, and nevirapine (ALN) regimen, whereas four patients (6.67%) were on the zidovudine, lamivudine, and lopinavir/ritonavir (ZL l/r) regimen. Almost an equal number of patients (3.33%) were on the abacavir, lamivudine, and efavirenz (ALE) and tenofovir, lamivudine, and efavirenz (TLE) regimens. Only one patient (1.67%) was on the tenofovir, lamivudine, and lopinavir/ritonavir (TL l/r) regimen (Figure 1).

The history of otitis media was present in only four (6.67%) out of 60 patients. Symptoms noted were earache in 18 (30.00%) cases, dizziness in eight (13.33%) cases, and tinnitus in seven (11.67%) cases.

The mean CD4 counts were 655.77 ± 301.13 cells/µL, and the mean viral load was 958.45 ± 2933.84 copies.

**Figure 2:**
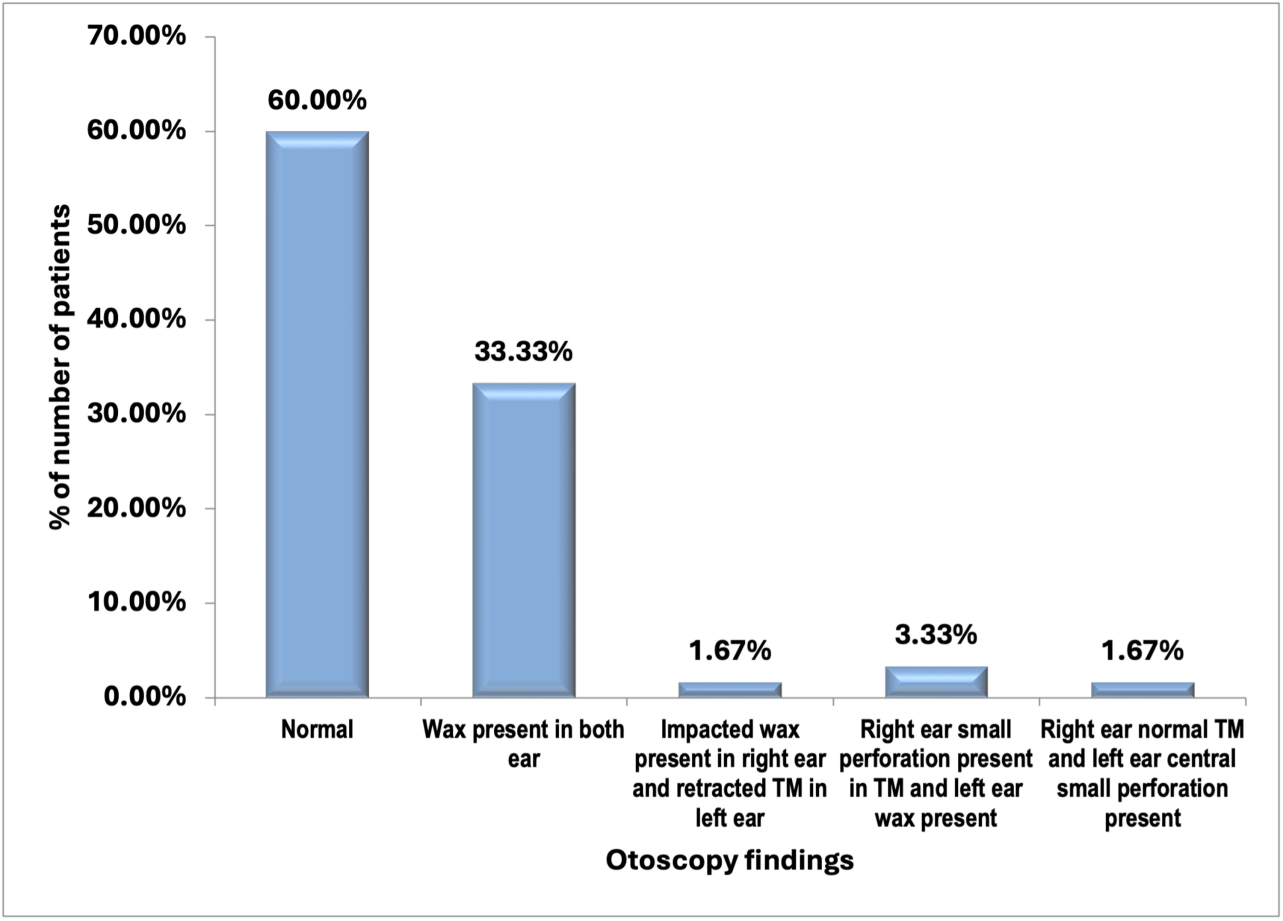
Distribution of otoscopy findings of the study subjects

In the majority (36 (60.00%)) of patients, otoscopy findings was normal, followed by wax present in both ears (20 (33.33%)) and right ear small perforation present in TM and left ear wax (two (3.33%)). Impacted wax was present in the right ear and retracted TM in the left ear and right ear normal TM and left ear central small perforation was present in only one out of 60 patients (1.67%) each (Figure 2).

HL was present in 10 (16.67%) cases, and all of them had CHL. Six cases had unilateral while four cases had bilateral HL. In seven cases, the degree of HL was mild while moderate HL was seen in two cases, and the degree of HL was minimal in only one out of 10 children.

Compared to patients without HL, patients with HL had comparable mean age (12.6 ± 2.59 years vs. 12.61 ± 3.03 years, p-value = 0.992); comparable gender distribution with 17.65% males and 15.38% females in HL+ group vs. 82.35% males and 84.62% females in the HL- group (p-value = 1); comparable mean age at diagnosis (4.8 ± 4.28 years vs. 5.18 ± 2.96 years, p-value = 0.733); and comparable mean duration of HIV (7.5 ± 2.88 years vs. 7.36 ± 3.26 years, p-value = 0.900). However, a significantly higher proportion of patients with HL had a history of otitis media (75% vs. 25%, p-value = 0.013). Mean CD4 counts were significantly lower in the HL+ group compared to the HL- group (483 ± 247.17 vs. 690.32 ± 301.09, p-value = 0.046). The median viral load was significantly higher in the HL+ group (2036.5 (1528-4010.25) vs. 0 (0-118.25), p-value < 0.0001) (Table 2).

**Table 2:** Association of hearing loss with patient characteristics and HIV disease severity HIV: human immunodeficiency virus; HL: hearing loss

Parameters	HL+ (n = 10)	HL- (n = 50)	P-value	Test used
Mean age (Mean + -SD)	12.6 ± 2.59	12.61 ± 3.03	0.992	Independent t-test
Gender, N(%)	Not applicable	Not applicable	1	Fisher's exact test
M	6 (17.65%)	28 (82.35%)	Not applicable	Not applicable
F	4 (15.38%)	22 (84.62%)	Not applicable	Not applicable
Mean age at diagnosis (Mean + -SD)	4.8 ± 4.28	5.18 ± 2.96	0.733	Independent t-test
Mean duration of HIV (Mean + -SD)	7.5 ± 2.88	7.36 ± 3.26	0.900	Independent t-test
H/o otitis media, N(%)	3 (75%)	1 (25%)	0.013	Fisher's exact test
CD4 counts (Mean+-SD)	483 ± 247.17	690.32 ± 301.09	0.046	Independent t-test
Viral load (Median, 25-75th interquartile range)	2036.5 (1528-4010.25)	0 (0-118.25)	<0.0001	Mann Whitney test

**Figure 3:**
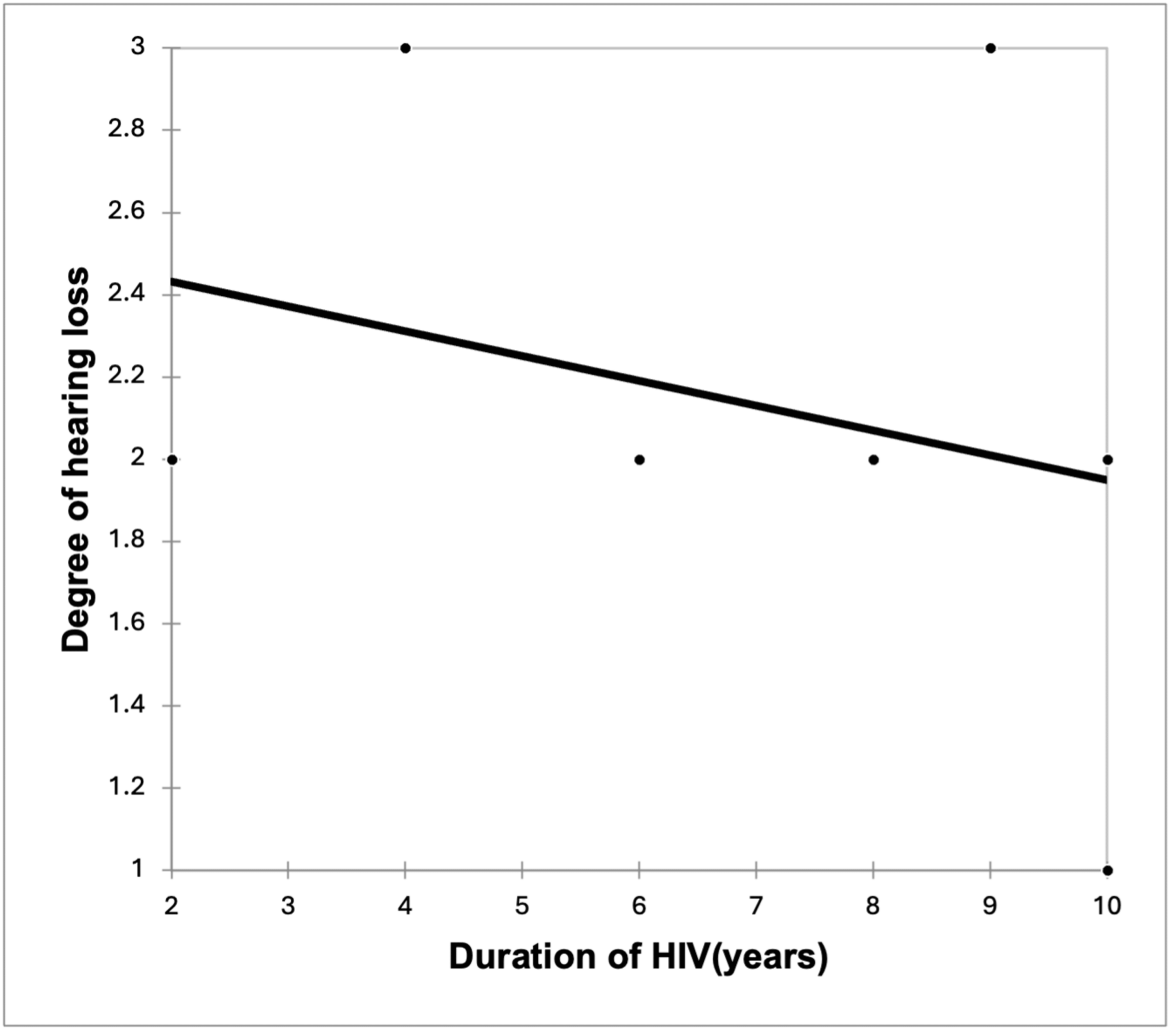
Correlation of duration of HIV (years) with the degree of hearing loss (r = -0.395, p = 0.261)

**Figure 4:**
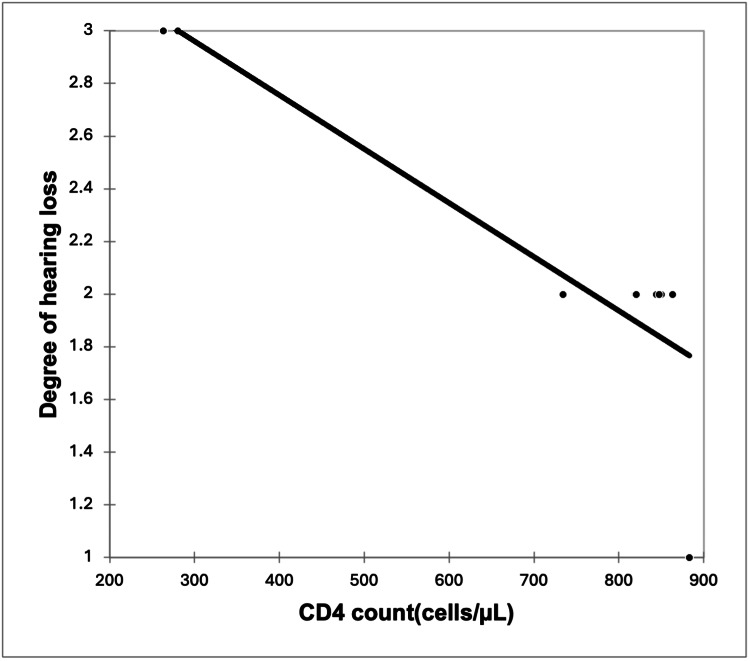
Correlation of CD4 count (cells/µL) with the degree of hearing loss (r = -0.809, p = 0.008)

**Figure 5:**
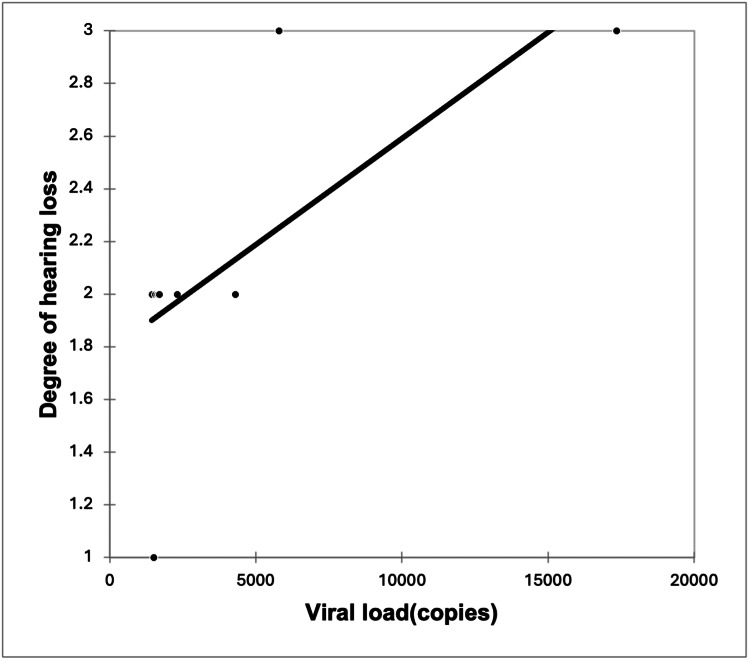
Correlation of viral load (copies) with the degree of hearing loss (r = 0.749, p = 0.018)

A significant strong positive correlation was seen between the severity of HL with viral load with a correlation coefficient of 0.749. A significant very strong negative correlation was seen between the severity of HL with CD4 counts with a correlation coefficient of -0.809. A non-significant weak negative correlation was seen between the severity of HL with the duration of HIV with a correlation coefficient of -0.395 (Figures 3-5).

## Discussion

We report an incidence of 16.67% HL among children with HIV. The findings are in line with the studies by Torre et al. [13] with a prevalence rate of 20% in 145 HIV-infected children; Devendra et al. [14] with a prevalence rate of 12% in 296 children with HIV; Maro et al. [15] with a prevalence rate of 17% in 97 children. In other studies, a higher rate was reported. Vincent et al. [2] found a 36.5% prevalence rate of HL in 200 HIV-positive children; Chao et al. [12] found that HL was present in 38.8% of HIV-infected children; and Christopher et al. [16] mentioned HL in 33% of children (n = 370). The variation may be due to heterogeneous populations, severity of HIV, and different durations of HIV. Moreover, it needs to be mentioned here that this only shows the association of HIV with HL, and in no way should it be interpreted as causation since the study was cross-sectional in design. 

Furthermore, it must be discussed here that the incidence of HL in HIV-positive children falls equivalent to HL in adults whose values vary between 14% and 49% [3].

All cases of HL had CHL in our study, which was consistent with the findings of the otoscopic examination that detected tympanic membrane (TM) perforation and impacted wax in patients with HL. The strong association of TM perforation with HL suggests a middle ear pathology, which is a significant contributor of HL in HIV patients [1-3].

Otitis media is frequently identified as a cause of ear infections in HIV-positive patients, and otitis media with effusion is also common. In this study, both conditions were found in a significant number of HIV-positive patients. Additionally, the impact of secondary opportunistic infections, which are common in individuals with HIV, as well as the use of ototoxic medications like Efavirenz, Tenofovir, or the virus directly affecting the central nervous system, peripheral nerves, vestibulocochlear nerve, or the cochlea, should not be underestimated [1,2]. In our study, there was no case of SNHL, which might be because of the small sample size or the fact that only pure-tone audiometry was done and otoacoustic emissions or auditory brainstem response testing was not used, which could have led to the missing cases of SNHL specifically.

As for the severity, we found that six cases had unilateral and four cases had bilateral HL.

Among other studies, Palacios et al. [17] found that unilateral HL was present in 67% of children and bilateral HL in 33% of children. Khoza-Shangase et al. [18] mentioned that unilateral and bilateral HL was present in 35% and 65% of children, respectively. Chao et al. [12] observed that unilateral HL was present in 48% of children and bilateral HL in 52% of children. In the study by Hrapcak et al. [19], most of the children (60%) had unilateral HL. Torre et al. [20] reported that 52% of children had unilateral HL and 48% had bilateral HL.

Primarily, the HL was mild in nature in seven cases, minimal in one case, and moderate in two cases.

Among other studies, Vincent CM et al. [2] reported that mild HL was present in 53 (72.6%) children, moderate in 18 (24.7%), and severe in two (2.7%) children. Hrapcak et al. [18] reported that mild HL was present in 66.7% of children, moderate in 20.6%, severe in 7.1%, and profound HL in 5.6% of children. Christopher et al. [16] found that mild, moderate, and severe HL was present in 36%, 58%, and 6% of children, respectively. Palacios et al. [17] found that mild HL was present in 25% of children and moderate HL in 75% of children.

It must be stressed here that the severity of HL showed a direct correlation with the viral load (r = 0.749, p = 0.018) and lower CD4 counts (r = -0.809, p = 0.008). With the increase in viral load, there was a significantly higher HL, and consequently, there was a fall in CD4 counts, indicating that with the severity of HIV, HL severity also increases. The association between HL and low CD4 cell count may be due to the accumulative damage to the TM and middle ear by recurrent episodes of otitis media, which was present in our study in patients with HL [18].

This was in line with the study by Chao CK et al. [12], who also observed that HL showed a significant correlation with lower CD4 cell count <500 cells/mm^3^, with an OR of 3.53 and P-value of 0.02. Vincent CM et al. [2] also reported similar findings as HL was significantly associated with lower CD4 count in HIV-positive children as compared to HIV-negative children with HL (682.15 ± 409.09 vs. 851.93 ± 476.24, P = 0.043). Palacios et al. [18] compared general patients with HL and HIV-positive children with HL, and it was found that an association of HL was seen with a lower absolute CD4+ count (209 vs. 620 cells/mL, P = 0.08) and higher viral load during audiological assessment (2239 copies of - viral RNA/mL vs. 1203 copies of viral RNA/mL, P = 0.22); however, statistical significance did not exist.

CD4 count levels indicate an individual's immune system strength, with lower levels suggesting weaker immunity. The extent of immunosuppression helps in the determination of the likelihood of opportunistic infections in HIV-positive children [3]. A high viral load can lead to HL due to opportunistic infections or a direct effect of the virus itself, and a high viral load also signifies non-compliance of the highly active antiretroviral therapy (HAART) [3].

However, we found that the duration of HIV had no significant correlation with HL severity, although there was a negative correlation with an r-value of -0.395, but the P-value was not significant, i.e., 0.261. This may be because we only specifically chose the children population. Longer periods of HIV infection would lengthen the time at risk for recurrent middle ear infections leading to HL.

These findings emphasize the importance of regular audiological assessment and management of HIV patients and the crucial need for communication rehabilitation services to decrease the impact of hearing impairment. Overall, the study holds strength in determining the association of HL in the early age of childhood infected with HIV, and this shows that children must be examined for hearing and appropriate precautions should be taken to manage the severity of HIV and prevent subsequent HL.

Limitations

The study was limited as it was restricted to a single hospital. The reason for HIV was not determined among the children. Adults or healthy normal children without HIV as the control population were not included in the study. The confounding effects of factors such as socioeconomic conditions, ART drug side effects and nutritional status, and past ear infections were not studied. Lastly, the study design was cross-sectional in nature, and follow-up was not done on the children.

## Conclusions

We report a prevalence rate of 16.67% HL among children with HIV. HL was predominantly mild in nature, and all cases had CHL. HL holds a direct correlation with the increasing severity of HIV disease and subsequent falling of CD4 counts. Based on the results, it is recommended that all HIV-positive children must undergo routine audiologic screening to determine the HL and provide them with audiological aid. Moreover, future follow-up research studies should be done to find out the association between ART and HL.
